# Lineage tracing reveals a novel PDGFRβ^+^ satellite cell subset that contributes to myo-regeneration of chronically injured rotator cuff muscle

**DOI:** 10.1038/s41598-024-58926-7

**Published:** 2024-04-26

**Authors:** Ayelet Dar, Angela Li, Frank A. Petrigliano

**Affiliations:** Department of Orthopaedic Surgery, Keck School of Medicine, University of Southern California, Los Angeles, CA USA

**Keywords:** Muscle stem cells, Regeneration

## Abstract

Massive rotator cuff (RC) tendon tears are associated with progressive fibro-adipogenesis and muscle atrophy that altogether cause shoulder muscle wasting. Platelet derived growth factor β (PDGFRβ) lineage cells, that co-express PDGFRα have previously been shown to directly contribute to scar formation and fat accumulation in a mouse model of irreversible tendon and nerve transection (TTDN). Conversely, PDGFRβ^+^ lineage cells have also been  shown to be myogenic in cultures and in other models of skeletal muscle injury. We therefore hypothesized that PDGFRβ demarcates two distinct RC residing subpopulations, fibro-adipogenic and myogenic, and aimed to elucidate the identity of the PDGFRβ myogenic precursors and evaluate their contribution, if any, to RC myo-regeneration. Lineage tracing revealed increasing contribution of PDGFRβ^+^ myo-progenitors to the formation of GFP^+^ myofibers, which were the most abundant myofiber type in regenerated muscle at 2 weeks post-TTDN. Muscle regeneration preceded muscle atrophy and both advanced from the lateral site of tendon transection to the farthest medial region. GFP^+^/PDGFRβ^+^Sca-1^−^lin^−^CXCR4^+^Integrin-β1^+^ marked a novel subset of satellite cells with confirmed myogenic properties. Further studies are warranted to identify the existence of PDGFRβ^+^ satellite cells in human and other mouse muscles and to define their myo-regenerative potential following acute and chronic muscle injury.

## Introduction

The progressive rotator cuff (RC) muscle atrophy and fibro-adipogenic degeneration witnessed after chronic tendon injury is well-defined and remains one of the major barriers to successful restoration of function following RC repair. Such degenerative changes not only diminish the power of the myotendinous unit but also reduces its compliance, resulting in a high-tension repair that is less likely to heal^1,2^. Consequently, recent investigative efforts have been focused on defining the cellular and mechanistic pathways that contribute to this unique fibro-adipogenic dystrophy that is observed in clinical setting of massive or chronic RC injury.

Although muscle dystrophy and muscle atrophy both lead to muscle wasting, they are not the same. While muscular dystrophy is a genetic condition caused by mutations in genes that are responsible for maintenance of healthy skeletal muscle phenotype, tissue composition and function, muscle atrophy refers to the loss of muscle tissue that can often be reversed with therapy and exercise. Supporting this notion, several studies reported that RC muscular atrophy can be stopped in successfully repaired RC^3,4^. Initial atrophy of the cuff muscles is a well-known prognostic factor for the anatomical results following cuff repair^3,5^ and postoperative change of the atrophy can be used as a prognostic factor for structural healing after cuff repair^2^. Although RC atrophy is a major prognostic factor for anatomic and functional results following surgical repair, very little remains known about the mechanisms that regulate RC skeletal muscle myo-remodeling following chronic injury and even less than that is known about the identity of cells that contribute to myo-remodeling of injured adult RC skeletal muscle. The best characterized muscle-residing myogenic precursors are the satellite cells (SC), identified on anatomic localization under the basal lamina and outside the myofiber plasma membrane, and Pax7 nuclear expression^6^. Studies performed in a mouse model of RC tendon transection have confirmed that SC are transiently activated, but not depleted, over a long period of time during which the muscle progressively degenerates^7^. Other myogenic, non-SC subsets have been discovered in other types of murine muscles: PW1^+^Pax7^−^ progenitors^8^ and alkaline phosphatase^+^ pericytes^9^ were shown to contribute to the development and regeneration of mouse postnatal skeletal muscle but not to that of adult skeletal muscle, while Twist2^+^ interstitial cells exhibited limited myogenic differentiation and contributed to the formation of a specific type of myofibers in adult mouse soleus muscle^10^. Additionally, markers such as CD146^11,12^ and alkaline phosphatase^13^ were used to identify and distinguish myogenic muscle perivascular cells from SC in adult muscle tissue and to demonstrate the myo-regenerative features of these cells. A direct contribution of PDGFRβ lineage-derived myogenic precursors was demonstrated in a mouse model of cardiotoxin-induced acute injury, but the identity of these myogenic progenitors remained ambiguous^14^. Gene expression studies of PDGF receptors have shown that quiescent adult muscle stem cells and myoblasts preferentially express PDGFRβ over PDGFRα and the role of PDGF receptors in regulation of myogenesis was implicated in vitro using C2C12 mouse myoblast cell line^15^. However, given that prolonged cultures and various culture conditions have been shown to artificially induce PDGFRβ expression in mouse and human SC or myoblasts^16,17^, only genetic inducible fate mapping combined with phenotypic and functional identification studies can unequivocally reveal the identity of PDGFRβ^+^ myogenic cells, which have the potential to contribute to muscle regeneration.

Co-expression of PDGFRβ with either PDGFRα, Sca-1 or both typifies non-myogenic, fibro-adipogenic progenitors that directly contribute to fibro-adipogenesis of the RC^18^, other types of skeletal muscles^19,20^ and multiple organs such as lung, liver and kidney^21,22^. Since it is well established that SC do not express Sca-1^23^ and that co-expression of PDGFRβ and Sca-1 demarcate fibro-adipogenic, non-myogenic progenitors^20^, we hypothesized that a marker combination of PDGFRβ^+^Sca-1^−^ may define a novel subset of SC.

To investigate the existence of a novel, functionally myogenic PDGFRβ^+^ cell subset in the settings of chronic RC skeletal muscle injury, we used an inducible PDGFRβ-Cre genetic tracing approach in combination with a well-characterized mouse model of massive RC tear^24^. The inducible tracing technology enables to turn on the fluorescent labeling of PDGFRβ^+^ cells by tamoxifen (TAM) induced expression of green fluorescent protein (GFP) at any selected age of mice, allowing a way to distinguish between the contribution of postnatal PDGFRβ^+^ cells and adult PDGFRβ^+^ cells to myo-regeneration of injured RC. In double fluorescent inducible mice, TAM induces the irreversible replacement of red tdTomato (tdT) expression with that of GFP only in PDGFRβ^+^ cells^25^. We found that PGDFRβ lineage GFP^+^ myo-progenitors contributed to robust myogenesis of injured RC after tendon transection and denervation (TTDN) within 2 weeks after injury, which is the time point at which temporary myo-regeneration is detected^18^. We also confirmed that muscle regeneration preceded fibro-adipogenic differentiation, and that GFP^+^ myofibers responded to environmental cues and progressively atrophied in a muscle region-dependent manner within 6 weeks post-TTDN. Analysis of marker expression revealed the existence of a novel GFP^+^PDGFRβ^+^lin^−^CXCR4^+^Integrin-β1^+^ SC subset and functional analysis of sorted GFP^+^ SC confirmed their myogenic features in cultures.

## Results

### PDGFRβ-cre recombination marks perivascular and interstitial cells in RC skeletal muscle

**Figure 1:**
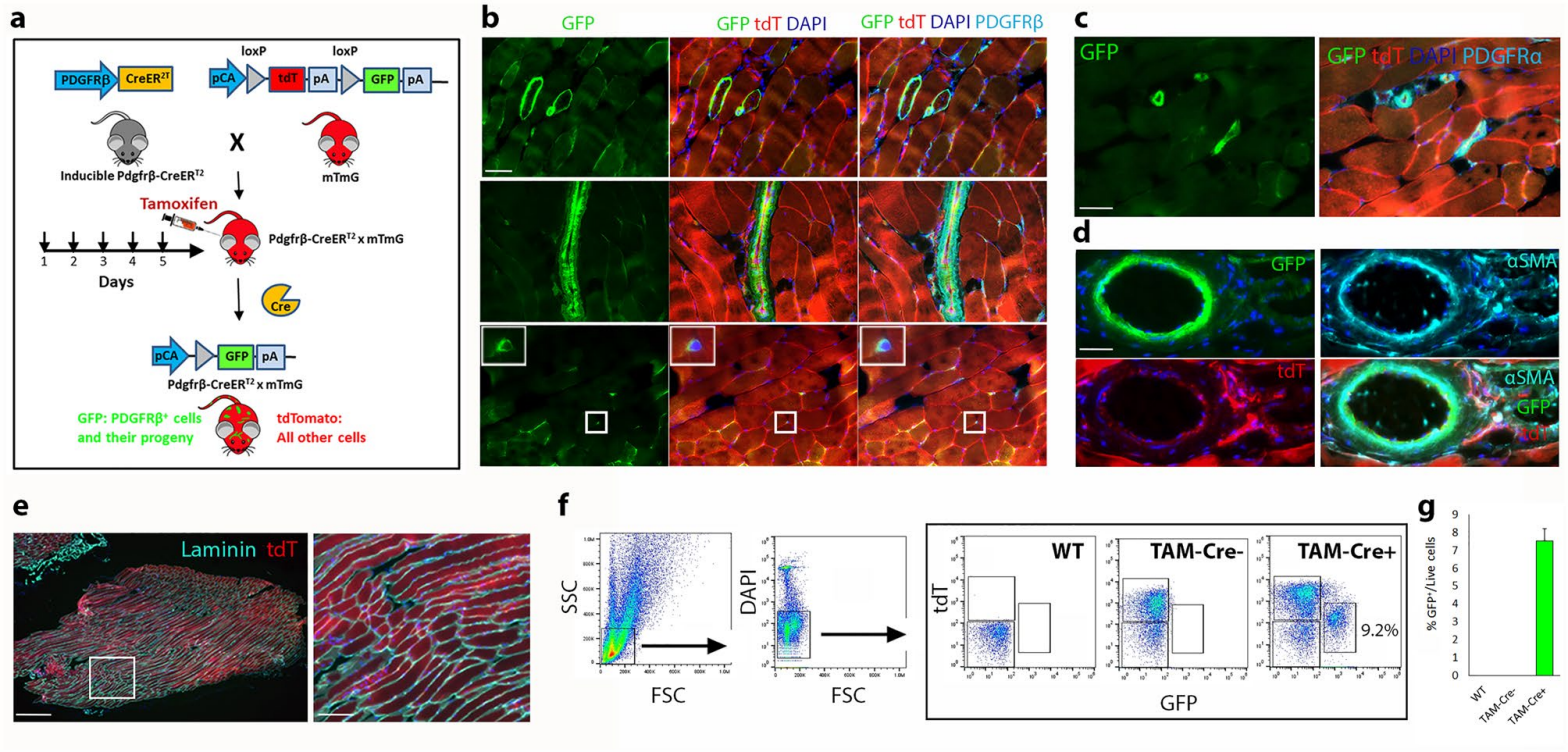
PDGFRβ expression in mouse adult RC skeletal muscle of inducible Pdgfrβ-CreER^+/−^;mTmG double fluorescent reporter mice. Before the administration of tamoxifen (TAM) for 5 consecutive days, all cells of Pdgfrβ-CreER^+/−^;mTmG offspring mice express only membrane-associated tandem dimer Tomato (tdT, red). After TAM administration, Cre mediates irreversible excision of tdT only in PDGFRβ-expressing cells, replacing it with constitutive expression of green fluorescent protein (GFP, green) and thereby enabling exclusive fate tracing of GFP^+^PDGFRβ^+^ cells and their progeny (green). pA—polyadenylation sequence. pCA—CMV enhancer/chicken beta-actin core promoter (**a**). Immunofluorescence analysis of PDGFRβ expression (cyan) revealing similar distribution with GFP (green) expression (**b–d**) in interstitial spaces between tdT^+^ (red) myofibers. Higher magnifications show GFP^+^ cells localized in the endomysium (**b**) and blood vessel residing GFP^+^ perivascular cells, identified by co-expression of either PDGFRα (cyan, **c**) or α-smooth muscle actin (αSMA, cyan, **d**). Laminin immunoreactivity (cyan, **e**) outlined individual tdT^+^ myofibers. All myofibers in sections of TAM-Cre^+^ mice maintained tdT expression and did not express GFP (**e**). Blue nuclei staining by DAPI (**b-e**). Representative dot plots of tdT and GFP expression by RC cells of wild type (WT) C57BL/6 (tdT^−^GFP^−^), TAM-Cre^−^ (tdT^+^GFP^−^) and TAM-Cre^+^ (tdT^+^GFP^+^) mice (**f**). Frequency of GFP^+^ cells in non-injured RC was analyzed by flow cytometry (**g**). Data are mean ± SEM. n = minimum of 3 mice per strain. Scales bars are 50 μm (**b,c,d**), 500 μm (**e**, left panel), and 100 μm (**e**, right panel).

In order to track PDGFRβ^+^ cells and their progeny in RC muscle, we crossed Pdgfr-β-CreER^T2^ mice with mTmG^25,26^ double-fluorescent reporter mice, which, following administration of TAM, express membrane-targeted green fluorescent protein (mGFP) in place a of membrane-targeted tandem dimer tomato (tdT) red fluorescent protein after Cre-mediated excision of the tdT locus in a given cell type (Fig. 1a). In uninjured RC, PDGFRβ-Cre induced expression of GFP, matched the expression of PDGFRβ detected in interstitial cells and blood vessels (Fig. 1b and Supplementary Fig. S1). Additionally, GFP co-expressed with PDGFRα (Fig. 1c and Supplementary Fig. S1) and α smooth muscle actin (Fig. 1d), which demarcate the perivascular cell layers and smooth muscle cells of blood vessels. Histological examination of axially sectioned RC muscles of TAM-induced Pdgfrβ-CreER^+/−^;mTmG (TAM-Cre^+^) mice confirmed that following TAM administration, all RC laminin^+^ myofibers retained tdT signal and did not express PDGFRβ. Moreover, the expression of GFP signal was not induced in any of non-injured RC myofibers of TAM injected mice (Fig. 1e and Supplementary Figs. S1 and S2). Notably, laminin staining exposed the unique alignment of myofibers in axially sectioned RC muscles. Unlike other skeletal muscles such as those in the hindlimbs^27^, where the myofibers are aligned in the same orientation, the myofibers of mouse RC are aligned in a mixed orientation (Fig. 1e). Finally, flow cytometry was used to further validate the specificity of Cre-recombination (Fig. 1f and Supplementary Fig. S2). Dot plot analysis of fresh single cell suspensions from uninjured RC of TAM-Cre^+^ mice confirmed that these cells expressed only GFP or tdT but not both (Fig. 1f). GFP^+^ subpopulation comprised 8.2 ± 1% out of DAPI^−^ live RC cells (Fig. 1g). RC of wild type C57BL/6 mice (GFP^−^tdT^−^ cells) as well as TAM-Cre^−^ mice (GFP^−^tdT^+^ RC cells) served as controls for all FACS and immunohistochemistry studies (Fig. 1f,g and Supplementary Fig. S2). It was previously demonstrated that muscle residing PDGFRβ^+^ mesodermal progenitors directly contribute to the progressive fibro-adipogenic degeneration of chronically injured mouse RC^18,28^. However, the direct contribution of PDGFRβ^+^ cells to chronic myogenic remodeling of mouse adult RC skeletal muscle has not yet been studied. Demonstrating the usability of TAM-induced Pdgfrβ-CreER;mTmG mice to study the role of PDGFRβ lineage in RC skeletal muscle remodeling (Fig. 1 and Supplementary Fig. S2), we next combined inducible genetic fate tracing analysis with a mouse model of chronic muscle injury induced by irreversible tendon and nerve transection (TTDN), to study the direct contribution GFP^+^PDGFRβ^+^ progenitors to myo-regeneration of adult RC skeletal muscle and define their identity.

### Irreversible nerve and tendon injury of RC induces temporary myo-regeneration followed by chronic muscle atrophy spreading from lateral region to the medial region of the muscle

**Figure 2:**
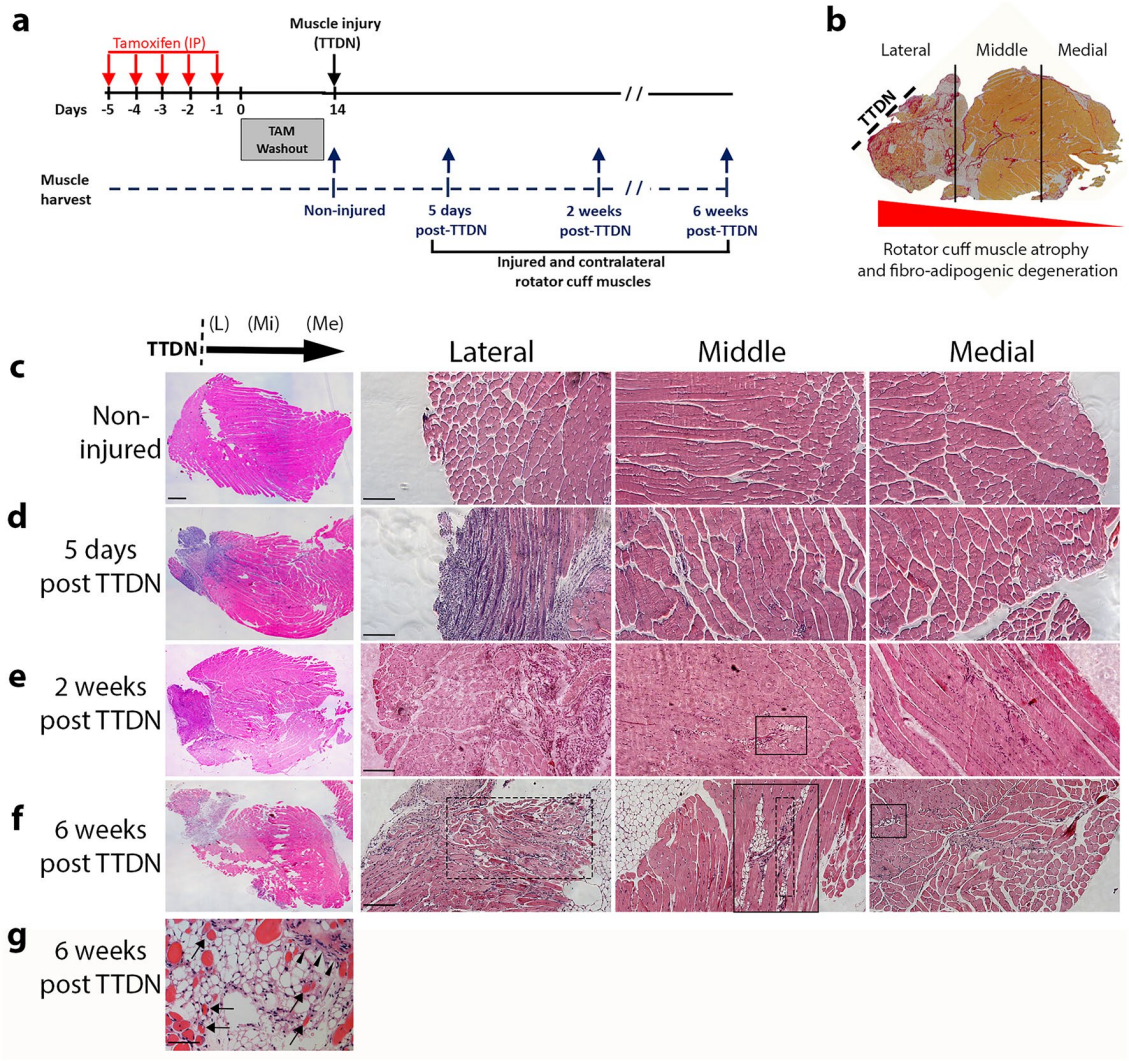
Kinetics of RC degenerative remodeling in TAM-injected inducible mice following massive RC tear surgery. Schematic representation of TAM-dependent induction of Cre activity and adult skeletal muscle RC injury in 3–5 month-old TAM-injected CreER^−/+^;mTmG (tdT^+^GFP^+^) mice (**a**). Muscle wasting and development of fibro-adipogenic damage progress from lateral site of tendon and nerve transection (TTDN) through the middle region to the farthest medial region (**b**). Representative H&E images of non-injured RC (**c**) and myogenesis in injured RC skeletal muscle at early- (5 days, **d**), intermediate- (2 weeks, **e**) and late- (6 weeks, **f**) stages of chronic RC remodeling. TTDN injury rapidly induces myofiber necrosis and increased cellularity within 5 days (**d**). A temporary regenerative myogenesis is seen at 2-weeks post-TTDN (**e**). Massive degenerative muscle atrophy develops in the lateral region at 6 weeks post-TTDN, detected to a lesser extent in the middle region (dashed squares, **f**), and is not observed in the medial region. Fibro-adipogenesis develops at 2-weeks post-TTDN and gradually progresses from the lateral region to medial region in 6-week injured RC (squares, **e**,**f**). Representative high magnification image showing atrophied myofibers (arrows), fibrotic scar (arrowheads), and accumulating pathological fat tissue (white area) that replace healthy muscle tissue within 6 weeks post-TTDN (**g**). Scale bars are 500 μm (**c–f**, left panels), 200 μm (**c–f**, lateral, middle, medial panels) and 50 μm (**g**).

Differing from experimental acute injuries that induce sustained muscle regeneration, massive RC tears induced by irreversible RC TTDN result in chronic and severe muscle atrophy, fibrosis and fatty infiltration^24,29^. We applied in the TAM-induced Pdgfrβ-CreER;mTmG mouse strain the TTDN model and confirmed the kinetics and direction of damage spreading at 5-days, 2- and 6-weeks following induction of injury (Fig. 2a). As expected^18,24,30,31^, massive muscle wasting, fibrosis and fat accumulation were seen within 6 weeks following TTDN, and the pathologic changes progressively advanced from the lateral site of transection through the adjacent middle region to the medial region far from the transection site (Fig. 2b). In addition, and coinciding with previous studies^18,30–32^, H&E staining confirmed that in comparison to non-injured RC (Fig. 2c), muscle cellularity was increased and myofiber necrosis was detected at the lateral region of the RC at 5 days post-TTDN, wherein the middle and medial regions remained unaffected (Fig. 2d). As expected, a temporary muscle regeneration coupled with the development of small adipocyte colonies was observed in the lateral and middle regions of the RC at 2 weeks post-TTDN (Fig. 2e). Degeneration of the injured RC built up with time, and at 6 weeks after TTDN, was associated with massive muscle atrophy and an increase in the size of muscle areas occupied by fibrotic scars and adipocytes (Fig. 2f,g). Muscle atrophy and fat accumulation were greater in the lateral region compared to the middle region, and only a few adipogenic clusters were detected in the medial region (Fig. 2f).

### Chronic injury of RC skeletal muscle induces progressive activation of PDGFRβ myogenic lineage

**Figure 3:**
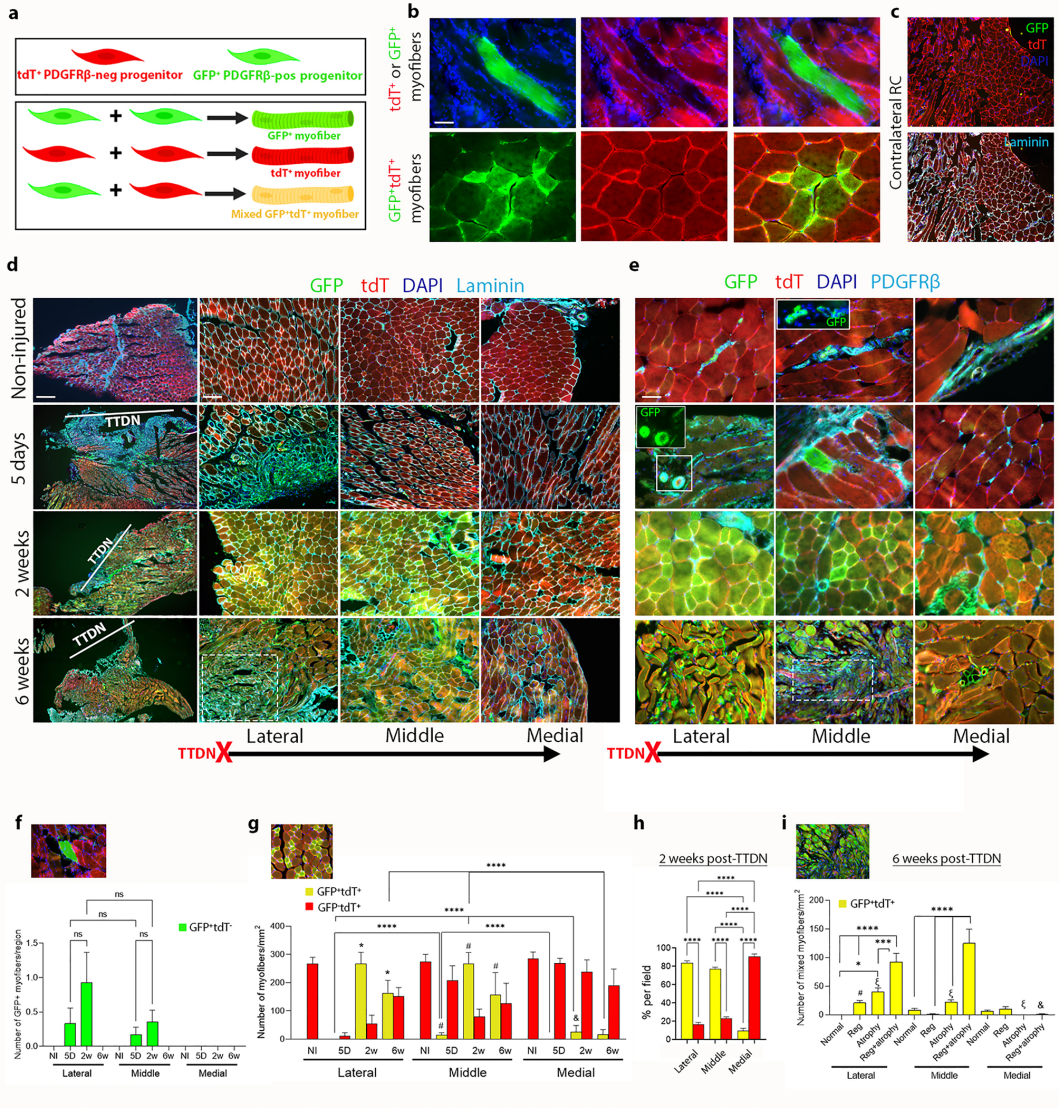
Myogenic GFP^+^PDGFRβ lineage cells markedly contribute to regeneration of myofibers following massive RC injury. Illustration of alternative fusion events between PDGFRβ^+^ and PDGFRβ^−^ myo-progenitors, which are reflected by the existence or absence of overlaps between tdT and GFP signals (**a**). Examples of regenerating (central DAPI^+^ nuclei) or newly formed GFP^+^tdT^−^, GFP^+^tdT^+^ and GFP^−^tdT^+^ myofibers in TTDN injured RC (**b**). Representative images of contralateral RC at either 5 days, 2- and 6-weeks post-TTDN. GFP signal was not detected in non-injured contralateral RC at any time point of muscle harvest (**c**). Laminin (**d**) or PDGFRβ (**e**) staining and quantifications of GFP^+^tdT^−^ (inset, **f**), GFP^+^tdT^+^ (inset, **g**) and GFP^−^tdT^+^ (insets, **f–g**) myofibers (**f-i**) in non-injured adult RC of TAM-Cre^+^ mice at the lateral, middle, and medial regions at the indicated time points post-TTDN (**d-i**) and at 2- (**h**) and 6- (**g**) weeks post-TTDN. Dashed square (**e**) and inset (**i**) show area of atrophied myofibers. Data (mean ± SEM) analyzed via one-way ANOVA and Šídák's post-hoc multiple comparisons. n = at least 3 mice per time point. * *P* < 0.05, *** *P* < 0.001, **** *P* < 0.0001, * *P* < 0.0001 between mixed myofibers in lateral region except for non-injured (NI) versus 5D, # *P* < 0.01 between regenerating (central nuclei, Reg+ atrophy) mixed myofibers in lateral and middle regions, & *P* < 0.01 between regenerating atrophied (central nuclei/atrophied, Reg) mixed myofibers in lateral and middle regions and in lateral and medial, ξ *P* < 0.01 between atrophied (peripheral nuclei/atrophied, Atrophy) mixed myofibers in all regions (Single factor analysis of variance). Scales bars are 50 μm (**b,e**), 500 μm (**c**, left panels), and 200 μm (**c**, middle and right panels).

Next, we analyzed the contribution of GFP^+^PDGFRβ^+^ cells to the myogenic remodeling of adult skeletal muscle in sections of non-injured as well as injured and non-injured contralateral RC of TAM-Cre^+^ mice at 5 days, 2- and 6-weeks following TTDN. To determine whether PDGFRβ^+^ cells contribute to muscle regeneration by initiating myogenesis autonomously or by fusing with tdT^+^PDGFRβ^−^ myogenic cells, we preformed histological analysis and classified myofibers into three types: (1) PDGFRβ lineage derived, green GFP^+^ myofibers, (2) PDGFRβ^−^ lineage derived, red tdT^+^ myofibers, and (3) all shades of yellow, mixed GFP^+^tdT^+^ myofibers derived from both PDGFRβ^+^ and PDGFRβ^−^ lineages (Fig. 3a,b). Between 3 and 5 months of age, GFP signal was not detected in laminin^+^ myofibers of non-injured contralateral RC that were harvested at 5-days, 2- and 6- weeks post TTDN (Fig. 3c and Supplementary Fig. S3) as well as non-injured adult RC (Fig. 3d–g) and all myofibers of these muscles expressed only tdT (Fig. 3d–g). These findings indicate that PDGFRβ lineage does not contribute to myofiber turnover of adult skeletal RC muscle between 3 and 5 months of age. Examination of TTDN-injured RC muscle sections revealed the presence of rare new regenerating green GFP^+^ myofibers only in the lateral and middle regions of injured RC at 5 days and 2 weeks post-TTDN (Fig. 3b,e,f and Supplementary Fig. S3). Massive necrosis of myofibers accompanied with higher labeling of GFP^+^PDGFRβ^+^ cells was observed at the lateral side within 5 days post-TTDN, while newly formed mixed laminin^+^GFP^+^tdT^+^ myofibers were detected in the middle region (Fig. 3d,g). The numbers and percentages of newly formed mixed GFP^+^tdT^+^ myofibers per field robustly increased within 2 weeks post-TTDN in the lateral and middle regions (Fig. 3d,e,g,h and Supplementary Fig. S3) and were significantly higher (*P* < 0.0001) than that of tdT^+^ fibers (Fig. 3g,h), implying for a greater myo-regenerative capacity of PDGFRβ lineage-derived myogenic precursors in the settings of chronic RC injury. PDGFRβ staining showed that all types of myofibers lacked its expression at all tested time points post-TTDN and that only blood vessels and interstitial cells exhibited overlapping high GFP expression (Fig. 3e). Altogether, these findings expose the existence of a PDGFRβ^+^ myogenic lineage that is profoundly activated in adult RC mouse muscle, fuse with other types of tdT^+^ PDGFRβ^−^ myo-progenitors and initiate myogenesis autonomously to much lesser extent.

Atrophy of the torn RC is concurrent with muscle degeneration and can be recognized on injured muscle sections by the reduction in diameter of myofibers. The averaged myofiber cross-section diameter in non-injured RC muscle was 80 ± 2.7 µm, (n = 3 mice, at least 15 sections per mouse, total of 100 myofibers) and was set as the threshold diameter of non-atrophic myofibers. The number of mixed GFP^+^tdT^+^ atrophied myofibers per field robustly increased within 6 weeks after injury, which is the late stage of RC remodeling that is associated with massive muscle wasting (Fig. 2b,f). It was previously shown that both non-regenerating (peripheral nuclei) and regenerating (central nuclei) atrophied myofibers are present in massively degenerated mouse RC at 6 weeks post-TTDN^31^. In accordance, both types of mixed GFP^+^tdT^+^ atrophied myofibers were seen in injured RC of TAM-Cre^+^ mice with significantly more regenerating mixed GFP^+^tdT^+^ atrophied myofibers in the lateral and middle regions in comparison to the medial region (Fig. 3i).

### PDGFRβ marks a novel subset of functionally myogenic satellite cells

**Figure 4:**
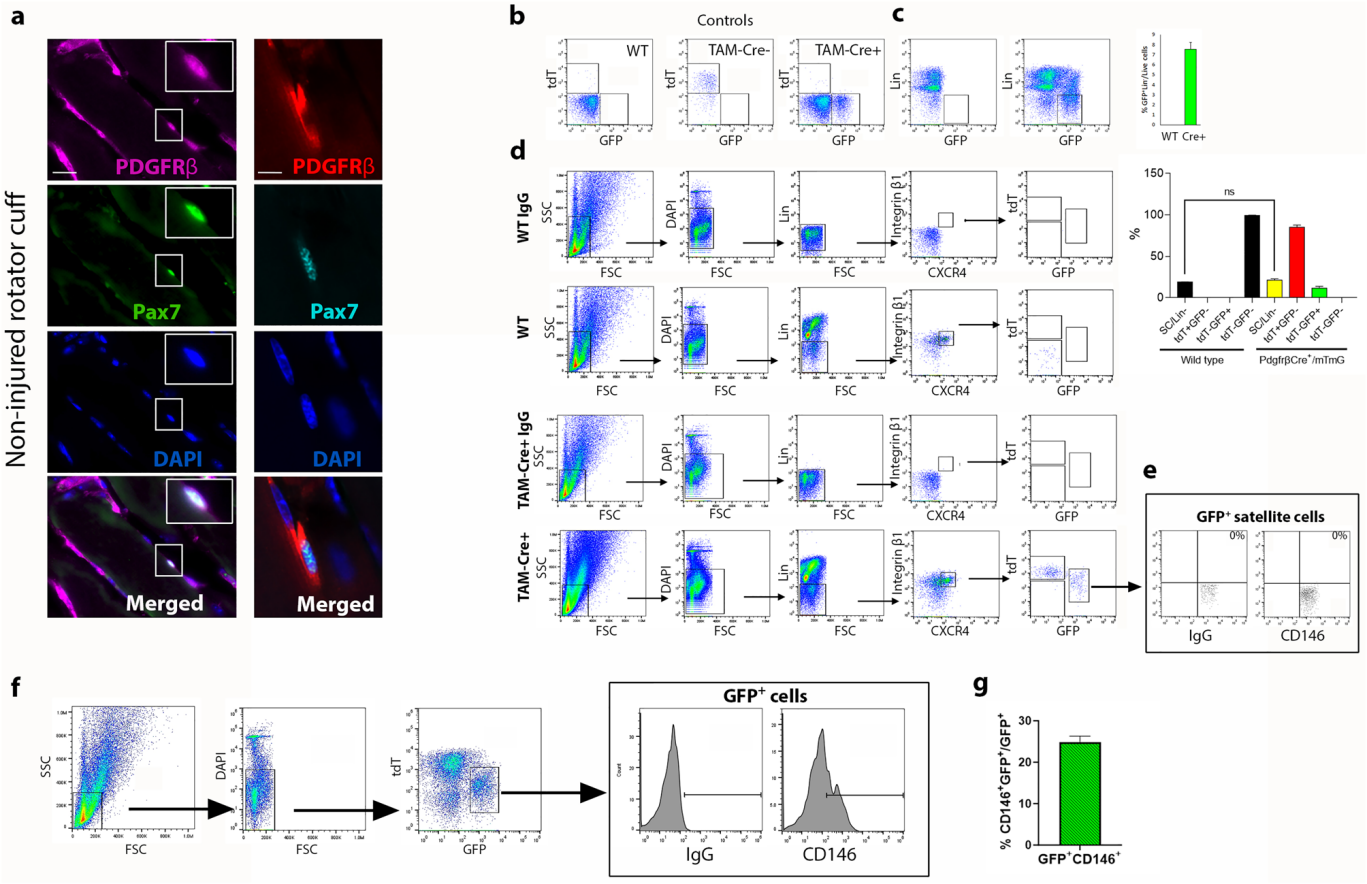
GFP^+^Lin^−^CXCR4^+^Integrinβ1^+^ typifies a novel subset PDGFRβ lineage-derived satellite cells in mouse RC. Two different clones of PDGFRβ antibodies, Y92 (left panel) and E28 (right panel), co-stain rare PDGFRβ^+^Pax7^+^ cells in sections of non-injured RC of 5-month-old C57BL/6 wild type mice. DAPI staining (blue) confirmed that Pax7 co-localized in the nucleus of the PDGFRβ^+^ satellite cell (**a**). Representative FACS analysis of mononuclear cells from non-injured RC of either C57BL/6 wild type (WT), TAM-Cre^−^ and TAM-Cre^+^ mice. WT, TAM-Cre^−^ and CD90-FITC stained WT cells were used as unlabeled and single controls for FACS analysis respectively (**b**). Representative dot plots and frequency of Lin^−^GFP^+^ cells in non-injured TAM-Cre^−^ and TAM-Cre^+^ RC cells. Lin indicates cell surface markers that are not expressed by satellite cells, including CD31, CD45, CD11b and Sca-1. Data are mean + SEM with 3 mice per group (**c**). Frequencies and analysis of tdT and GFP expression by Lin^−^CXCR4^+^Integrin-β1^+^ satellite cells (SC) of WT and TAM-Cre^+^ RC. DAPI^+^ dead cells were gated out. IgG indicates matched isotype control antibodies. Data (mean ± SEM, 3 mice per group) analyzed via one-way ANOVA and Šídák's post-hoc multiple comparisons. ns – non-significant, single factor analysis of variance (**d**). Representative dot plots of FACS analysis (n = 4 mice) showing that expression of CD146 is not detected in GFP^+^ SC (**e**). FACS analysis (**f**) and frequencies (**g**) of CD146 expression in GFP^+^ RC cells (n = 3 mice). Scale bars are 20 μm.

Sca-1 has proven to be a reliable marker that can be used to distinguish between non-activated SC and all other muscle cell types in the non-injured skeletal muscle^20,33^. While non-myogenic, fibro-adipogenic skeletal muscle progenitors express micro-heterogenous levels of Sca-1 that in turn regulate their fibro-adipogenic fate decision^34^, lack of Sca-1 expression combined with negative selection for lineage markers (lin) expressed by non-myogenic cells (CD31, CD45, CD11b, Ter119) and positive selection for β1-integrin and CXCR4 is used to identify and isolate Pax7^+^ SC^35,36^. Therefore, the following experiments were set up to determine whether or not myogenic PDGFRβ SC exist in RC skeletal muscle. Rare cells co-expressing PDGFRβ and nuclear Pax7 were found in sections of adult skeletal RC muscle of wild-type C57BL/6 mice between 3 and 5 months of age while interstitial PDGFRβ^+^Pax7^−^ cells were abundant in all RC sections (Fig. 4a). Due to the rarity of PDGFRβ^+^Pax7^+^ cells and for validation of staining reliability, we tested 2 different clones of PDGFRβ antibodies: Y92 and 28E. Similar frequency of antibody positivity of 1 ± 0.5 PDGFRβ^+^Pax7^+^ cells per section of the entire area of supraspinatus or infraspinatus muscles was quantified by fluorescent immunostaining (Fig. 4a). Additionally, microscopic analysis revealed that PDGFRβ^+^Pax7^+^ cells were randomly distributed throughout both supraspinatus and infraspinatus muscles. Next, flow cytometry analysis and cell sorting were used to validate that PDGFRβ^+^ myogenic cells exhibit phenotypic and functional SC properties. We first fractionated fresh DAPI^−^ live cells derived from RC of either WT or TAM-Cre^−^ and TAM-Cre^+^ mice based on the expression of GFP and Sca-1/lin markers. As expected GFP^+^ subset was detected only in TAM-Cre^+^ mice (Fig. 4b) and consisted of GFP^+^Sca-1^+^Lin^+^ and GFP^+^Sca-1^−^Lin^−^ subpopulations (Fig. 4c), implying for the potential existence of GFP^+^Sca-1^−^ myogenic SC. We also compared the frequencies of fresh SC in RC of TAM-Cre^−^ and matched wild-type mouse strain C57BL/6  (WT). We found that the frequency of Sca-1^−^lin^−^CXCR4^+^β1-integrin^+^ SC in TAM-Cre^+^ RC was similar to that of WT RC and reconfirmed that all SC of the WT RC were GFP^−^tdT^−^ (Fig. 4d). FACS analysis revealed that a significantly larger fraction of TAM-Cre^+^ SC expressed tdT only (85.2 ± 2) wherein a small fraction of these SC (11.7 ± 1.7) was derived from PDGFRβ lineage as indicated by TAM-induced GFP co-expression by Sca-1^−^lin^−^CXCR4^+^β1-integrin^+^ SC (Fig. 4d). FACS analysis confirmed that GFP^+^ SC did not express the mesodermal/perivascular cell marker CD146 (Fig. 4e) and immunohistochemistry confirmed that RC residing Pax7^+^ cells did not co-express CD146 (Supplementary Fig. S4). Coinciding with multiple other studies, demonstrating expression of CD146^11,37^ (Supplementary Fig. S4) or PDGFRβ in mouse^9,14,37^ (Fig. 1 and Supplementary Fig. S1) as well as human^12,13,17^ muscle myogenic perivascular cells, a fraction of RC muscle GFP^+^ cells (25 ± 0.8%) co-expressed CD146 (Fig. 4f,g).

### PDGFRβ^+^ SC-derived GFP^+^ myoblasts display higher fusion index than that of PDGFRβ^−^ SC-derived tdT^+^ myoblasts

**Figure 5:**
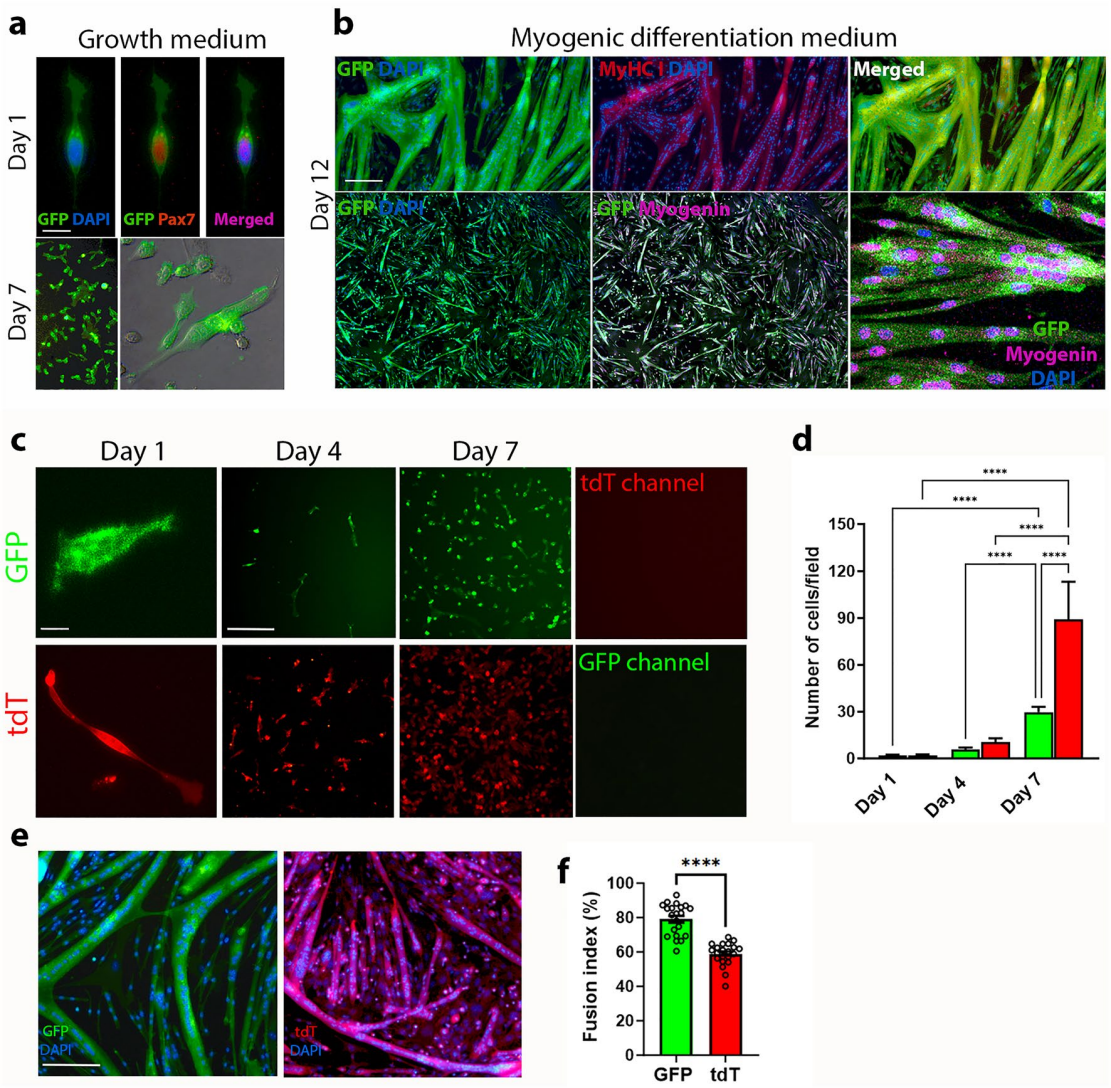
Myogenic potential of PDGFRβ^+^ SC in vitro. Representative images of PAX7 (red) and nuclear (DAPI, blue) staining of sorted GFP^+^Lin^−^CXCR4^+^Integrin-β1 cells (**a**). Expansion and sustained GFP expression of sorted GFP^+^ SC in 7-day-old cultures supplemented with growth medium (**a**). Myosin heavy chain I and (MyHC I, red) myogenin (magenta) and nuclear DAPI (blue) staining revealed that cultured GFP^+^ SC formed branched multinucleated GFP^+^ myotubes in differentiation medium, that uniformly expressed MyHC I and nuclear myogenin (**b**). Growth and myogenesis of sorted GFP^+^ SC and tdT^+^ SC in cultures (**c–f**). Sorted subsets were cultured in growth medium for 7 days (**c**) and viable cells were counted at the indicated timepoints (**d**). Data (mean ± SEM, 3 mice per group, cell subsets were seeded in triplicates) analyzed via one-way ANOVA and Šídák's post-hoc multiple comparisons. n = 3 mice, cell subsets were seeded in triplicates. **** *P* < 0.0001. Formation of GFP^+^ or tdT^+^ multinucleated myotubes in differentiation medium after induction of myogenesis for 3 days (**e**). Fusion index was calculated as the percentage of nuclei within multinucleated myotubes compared with the total number of nuclei in imaged field (**f**). Data are mean ± SEM. Two-sample t-test. n = 3 mice, cell subsets were seeded in triplicates. **** *P* < 0.0001. Scale bars are 20 μm (**a**,**c**, left panels), 100 μm (**b**,**e**) and, 200 μm (**c**, middle and right panels).

FACS sorted GFP^+^ SC that were fixed within 12 h post sorting expressed nuclear Pax7, an established marker of SC (Fig. 5a). Moreover, freshly sorted GFP^+^ SC proliferated rapidly within 7 days post isolation, did not express tdT and retained high GFP expression (Fig. 5a). Within 3 days in differentiation medium GFP^+^ SC exhibited robust myogenic differentiation and formed GFP^+^ multinucleated myotubes. Additionally, GFP^+^ myotubes co-expressed Myosin Heavy Chain I and Myogenin (Fig. 5b), which are well established markers of terminally differentiated mature myotubes^38^. Due to the rarity of GFP^+^ SC, myogenic cultures of sorted GFP^+^SC and tdT^+^SC were established at a low initial cell seeding concentration of 170 SC/cm^2^. Viable cell counts (except for DAPI-based count of PFA-fixed, tdT^+^ SC, following 7 days in culture due to high cell density) revealed that in the presence of growth medium, tdT^+^ SC proliferated faster than GFP^+^ SC, with a 2- and 3-fold increase in tdT^+^ SC numbers in comparison to that of GFP^+^ SC within 4 and 7 days in culture respectively (Fig. 5c,d). GFP signal was not detected in tdT cultures and tdT signal was not detected in GFP cultures after 7 days of cell cultivation (Fig. 5c), indicating for culture uniformity as well as stable expression of GFP and tdT. However, the fusion index of GFP^+^ myoblasts was significantly higher (P < 0.0001) than that of tdT^+^ myoblasts within 3 days of differentiation medium (Fig. 5e,f), implying for accelerated myogenic maturation of PDGFRβ^+^ SC-derived GFP^+^ myoblasts compared to PDGFRβ^−^ SC-derived tdT^+^ myoblasts.

## Discussion

Experimental chronic RC injury in the inducible Pdgfrβ-CreER;mTmG transgenic reporter mouse has allowed us to reveal the existence of a novel muscle residing myogenic PDGFRβ^+^ SC subset that is robustly activated and directly contribute to regeneration of adult RC skeletal muscle. Additionally, kinetic analysis of myogenesis in vivo demonstrates that injury-induced RC disease that begins with a primary RC tendon tear is followed by a secondary regenerative and degenerative myo-remodeling that develops in the RC musculature. The early (5 days post-TTDN) and intermediate (2 weeks post-TTDN) myo-remodeling stages of injured RC match and parallel the two first phases that have been identified in the process of muscle regeneration after acute injury: (1) necrosis of the injured muscle cell accompanied by increased cellularity of interstitial cells due to proliferation of the activated muscle progenitors and, (2) differentiation and fusion of myo-progenitors that form new muscle fibers^39^. However, the third late stage of RC myo-remodeling is completely different than that of acutely injured muscle. While myogenesis ends after muscle regeneration in the settings of acute muscle injury^39^ it is persistent in the fibro-adipogenic RC muscle at late stage of remodeling (6 weeks post-TTDN) as indicated by the presence of regenerating non-atrophied and atrophied fibers identified by the presence of a central nuclei. In experimental tendon and nerve injury, muscle degeneration is chronic and progressive in comparison to acute muscle injury. Consequently, pro-differentiation signals may last longer, through either constant SC turnover or replacement of ECM components by fibro-adipogenic PDGFRα^+^ cells or both, which in turn results in the creation of a microenvironment that favors activation of atypical myo-precursors as demonstrated here. Given that RC regenerative myogenesis precedes massive degenerative fibro-adipogenesis, it is most likely that replacement of ECM composition and increased collagen production^18,24^ deliver continuous stimuli of myogenic differentiation that leads to dysfunctional myogenesis and muscle atrophy. Considering that both subsets of Pax7^+^ SC, GFP^+^ and tdT^+^, were randomly distributed throughout RC muscle tissue, it is more likely that activation of SC myogenesis follows the damage progression induced by secondary muscle injury post-TTDN. Our finding that the kinetics of myo-regeneration matched the progression of muscle damage further supports this notion. Additionally, as suggested before^9,40^, SC may be required to induce the differentiation of other myogenic cell subsets as “upstream” cellular components in muscle myogenesis. The fact that the majority of RC derived myotubes exhibited a mixed tdT^+^GFP^+^ phenotype in vivo within 2 weeks post-TTDN strongly supports this assumption.

Recent studies prove the existence of adult muscle resident myogenic precursors distinct from SC, including vessel associated pericytes^9,37,41^, adventitial cells^42^, myogenic endothelial cells^43^ and, Twist2^+^ interstitial cells^10^. However, it is still unknown whether and when alternative progenitors may substitute for SC in the adult skeletal muscle. For example, while conditional ablation of Pax7^+^ SC has abrogated muscle regeneration^9,40,44,45^ and Pax7^+^ tracing studies revealed that SC labeled all types of muscle, Twist^+^ cells contributed exclusively to the formation of type IIb/x fibers^10^. Therefore, identification of PDGFRβ lineage myogenic cells as SC together with their robust activation and significant contribution to myo-regeneration predicts their superior capability to replenish and engraft the chronically injured RC. Further experiments should be conducted to evaluate the muscle engraftment and reconstruction ability of PDGFRβ^+^ SC following transplantation into remodeling RC and other chronically injured skeletal muscles.

In another model of denervation of rat extensor digitorum longus muscle, the SC population has been shown to increase during the first two months post denervation^46^, by that coinciding with another publication reporting a transient increase in the frequency of SC that was measured during the same time frame in a mouse model of RC tendon transection^7^. Both studies reported a pronounce decline in the frequency of SC at later time points^7,46^. We further demonstrate here a novel phenotypic heterogeneity among SC that in accordance can be further subdivided into two SC subpopulations based on PDGFRβ expression. Further studies of functional heterogeneity will reveal if in comparison to PDGFRβ^−^ SC subset, PDGFRβ^+^ SC possess different and/or superior myogenic properties in vivo and ex vivo.

Data linking between PDGFRβ protein expression and SC are so far implicit: while low levels of PDGFRβ RNA were detected in fresh SC isolated from 18-month-old mice, PDGFRβ was not detected at the protein level^47^. Our findings explain in part this discrepancy by demonstrating that PDGFRβ protein is not uniformly expressed by all SC and its detection is assay dependent. Another study demonstrated the direct contribution of PDGFRβ lineage myogenic cells to muscle regeneration^14^ but identification of these cells was performed using the pre-plating technique and long-term cultures, both are known for inducing expression of different markers than those observed in native tissues^16,17^. In order to overcome these limitations and to unequivocally prove that functional subset of PDGFRβ^+^ SC exist in RC muscle, we combined two complementary techniques: microscopy was used to directly observe PDGFRβ^+^Pax7^+^ SC in native RC muscle tissue and FACS analysis was used for identification and sorting of fresh cells isolated from non-injured RC based on the co-expression of GFP and characteristic SC markers. These in vitro findings confirmed that a seldom used chronic muscle injury model, applied in inducible PDGFRβ mice, revealed the existence of a novel myogenic PDGFRβ^+^ SC in mice. Identification in humans of equivalent PDGFRβ^+^ myogenic cells, exhibiting superior myogenic potential in pathologic conditions in which SC regenerative function is reduced or compromised, appears as a priority.

Clinical observations^48^ and murine experimental models of RC or hind limb muscle TTDN^49^ indicate that the RC is prone to massive fat accumulation. Other tendon tears (e.g. Achilles, quadriceps, distal biceps and triceps) may trigger significant muscle atrophy but rarely accumulate adipose tissue. Similarly, different rates of postnatal muscle regeneration by resident myogenic cells were measured in different murine muscles^9^ and more rapid atrophy was documented in denervated muscles of the rat hand, as compared to the arm^50^. Altogether these findings emphasize that different muscles can display unique pathophysiological responses that may lead to distinct phenotypes and characteristic clinical complications. Better understanding of the myogenic cellular composition and interactions as well as molecular pathways activated upon injury of different muscles will assist to adjust the relevant therapy per each.

In search of improvement of muscle regeneration by cell transplantation several successful investigational strategies have been developed including pre-conditioning of recipient muscle^51,52^, genetic manipulation of autologous myo-precursors^53^, and administration of myogenic cells that are derived from more accessible sources than muscle tissue, such as adipose or bone marrow tissue^37,54–57^ and pluripotent stem cells^31,53,58–60^. Still, in contrast to animal experimental studies, in which stage specific remodeling can be easily monitored for optimization of cell engraftment, it is very challenging and seldom possible to precisely control the best timing for transplantation in the clinic. Further research is required to evaluate the feasibility of PDGFRβ^+^ SC to replenish the RC and other muscles at later stages of degeneration.

## Materials and methods

### Mice

Pdgfrβ-CreER^T2^ mice, #029684, The Jackson Laboratory, were crossed with mTmG (tdTomato-EGFP) mice #007676, The Jackson laboratory^25^. C57BL/6 mice were used as Pdgfrβ-CreER mice matched wild type strain. Tamoxifen (TAM) injected Cre^−^ (Pdgfr-β-CreER^−/−^;mTmG) were used as control mice. Mice were genotyped between the ages of 4–7 weeks and both Pdgfrβ-CreER^+/−^;mTmG or Pdgfrβ-CreER^−/−^;mTmG male and female mice were used.

### Rotator Cuff injury

All animal procedures were approved by the USC Institutional Animal Care and Use Committee (IACUC) and all methods were performed in accordance with the relevant guidelines and regulations. This study was reported in compliance with the ARRIVE guidelines. Mice (8-week-old) were given 5 consecutive intraperitoneal injections of TAM (Sigma-Aldrich) for induction of recombinase (Cre) activity (80 mg/kg of body weight), followed by a washout period of 2 weeks. At the end of the TAM washout period mice underwent unilateral supraspinatus and infraspinatus tendon transection (TT) and irreversible denervation (DN) of the suprascapular nerve as previously described^24^.

### Histology, immunohistochemistry and immunocytochemistry

RC muscles were fixed in 4% formalin, embedded in paraffin, sectioned, dehydrated, and stained with H&E for general tissue structure. For fluorescence microscopy muscles were fixed with 4% paraformaldehyde (PFA) for one hour, washed in PBS, cryoprotected using a sucrose gradient (10%, 20% and 30%) and frozen in O.C.T compound (Tissue-Teck). Histological sections (12 μm thick) were prepared from frozen blocks using Leica CM 1860 UV cryostat. Sections of non-injured and contralateral RC of TAM-injected Pdgfrβ-CreER^+/−^;mTmG and Pdgfrβ-CreER^−/−^;mTmG mice were screened for GFP signal in myofibers (n = at least 10 RC per group with 25 sections per RC). For fluorescence microscopy sections or 4% PFA fixed cultures were incubated overnight at 4 °C with the primary antibodies, including rabbit-anti laminin (Sigma-Aldrich, 1:1000), rabbit-anti-PDGFRβ (1:100, Cell Signaling Technology), rabbit-ant-PDGFRα (1:500, Cell Signaling Technology), rabbit-anti-CD146 (1:100, Cell Signaling Technology), rabbit-anti-αSMA (Abcam, 1:100), mouse-anti-MyHC I (R&D Systems, 1:100), mouse-anti-Pax7 (1:40, DHSB) and rabbit-anti-myogenin (Invitrogen, 1:100). Alexa Fluor-conjugated secondary antibodies (1:400, one hour at RT, Invitrogen) were used for detection of primary antibodies and DAPI (1:1000, Molecular Probes) used for labeling of nuclei. Rabbit IgG and mouse IgG antibodies (Cell Signaling Technology) were used as IgG isotype controls at primary antibody matching concentrations. Images were acquired with a CKX53F3 Compact Cell Culture Microscope (Olympus, Oberkochen, Germany) and a Keyence BZ-X (Itasca, IL, USA).

### Flow cytometry and cell sorting

Supraspinatus and infraspinatus were harvested, enzymatically dissociated, and labeled for SC FACS analysis and sorting as described^23,27^. Briefly, Fresh RC cells were labeled with APC or PE conjugated antibodies against CD31, CD45, CD11b and Sca-1 (collectively referred to as “lin”) as well as APC-Cy7-anti-Integrin-β1 and biotin-anti-CXCR4. A secondary antibody, Streptavidin-PeCy7 (1:200) was used for CXCR4 detection. Immediately prior to analysis, 300 μl of DAPI was added (incubated for 3 min at RT and washed with PBS) to allow gating of dead cells. SC were defined as lin^−^CXCR4^+^Integrin-β1^+^. To allow gating of tdT^−^ and tdT^+^ populations, isolations were done in WT C57BL/6 (tdT^−^GFP^−^) and Pdgfrβ-CreER^−/−^;mTmG (tdT^+^GFP^−^) mice. C57BL/6 WT cells were stained with FITC-anti-CD90 to allow gating of GFP populations. Additionally, GFP^+^ cells were analyzed for co-expression of either PDGFRβ or CD146. Cell sorting was performed using FACS via an Aria III Flow Cytometer (BD Biosciences). Debris were excluded according to forward and side scatter data. Analyses were carried out using an Attune flow cytometer (Thermo Fisher Scientific) and FlowJo v10.8.1.

### Cultures and myogenic differentiation assay

GFP^+^ SC were cultured and induced to differentiate in myogenic cultures as previously described^27^ with modifications. Briefly, fresh GFP^+^tdT^−^lin^−^CXCR4^+^Integrin-β1^+^ sorted SC (n = 3 TAM-Cre^+^ mice) were seeded onto Matrigel coated dishes and cultured for 10 days in growth medium consisting of 20% horse serum, 1% Pen/Strep, and 1% Glutamine in F10 media, with daily addition of 5 ng/mL of bFGF. When cells reached confluency at 10 days post seeding, growth medium was changed to differentiation medium, consisting of 2.5% horse serum and 1% Pen/Step, and 1% glutamine in DMEM. Following 3 days in differentiation medium, cultures were fixed in 4% PFA and stained with either MyHC I or myogenin. DAPI was used for nuclear staining. Viable cell counting of sorted GFP^+^ SC and tdT^+^ SC (n = 3 mice, sorted cell subsets were seeded in triplicates) was performed at 1-, 4- and 7-days post cell seeding (170 SC/cm^2^). Due to high cell density of tdT^+^ SC after 7 days in growth cultures, only tdT^+^ cells were fixed in 4% PFA at 7 days post seeding and quantified based on nuclear staining by DAPI using ImageJ. The fusion index was calculated as the percentage of nuclei in fused myotubes (n ≥ 3 DAPI^+^ nuclei) out of the total nuclei in field.

### Quantification of myogenesis in RC skeletal muscles

Laminin-stained RC sections were imaged, and muscle fiber cross-sectional width was measured using Fiji ImageJ (National Institutes of Health). All laminin^+^ muscle fibers or muscle fibers outlined by membranal tdT within each imaged field were counted and classified based on overlap or lack of overlap between GFP and tdT signals. DAPI staining was used to visualize and count atrophied myofibers containing central or peripheral nuclei.

### Statistical analysis

All obtained data are presented as mean ± SEM. Statistical analysis was performed via GraphPad Prism software and using one-way ANOVA and Šídák's post-hoc multiple comparisons or two-sample t-test. *P* < 0.05 was considered statistically significant.

### Supplementary Information

Supplementary Information.

## Data Availability

The data used to support the findings of this study are included within the article and are available from the corresponding author on reasonable request.
