# Supplementary material for: Lineage tracing reveals a novel PDGFRβ+ satellite cell subset that contributes to myo-regeneration of chronically injured rotator cuff muscle
Source: Sci Rep. 2024 Apr 26;14:9668. doi: 10.1038/s41598-024-58926-7 (PMC11053018; doi:10.1038/s41598-024-58926-7)
Supplement: Supplementary file 1 — Supplementary Information. [file 41598_2024_58926_MOESM1_ESM.pdf]

Supplementary Material

Lineage tracing reveals a novel PDGFRβ$^+$ satellite cell subset that contributes to myo-regeneration of chronically injured rotator cuff muscle

Ayelet Dar[1]*, Angela Li[1], Frank A. Petrigliano[1*]

[1]Department of Orthopaedic Surgery, Keck School of Medicine, University of Southern California, Los Angeles, CA, United States.

***Correspondence:** darayelet@gmail.com, daroakni@usc.edu; Frank.Petrigliano@med.usc.edu

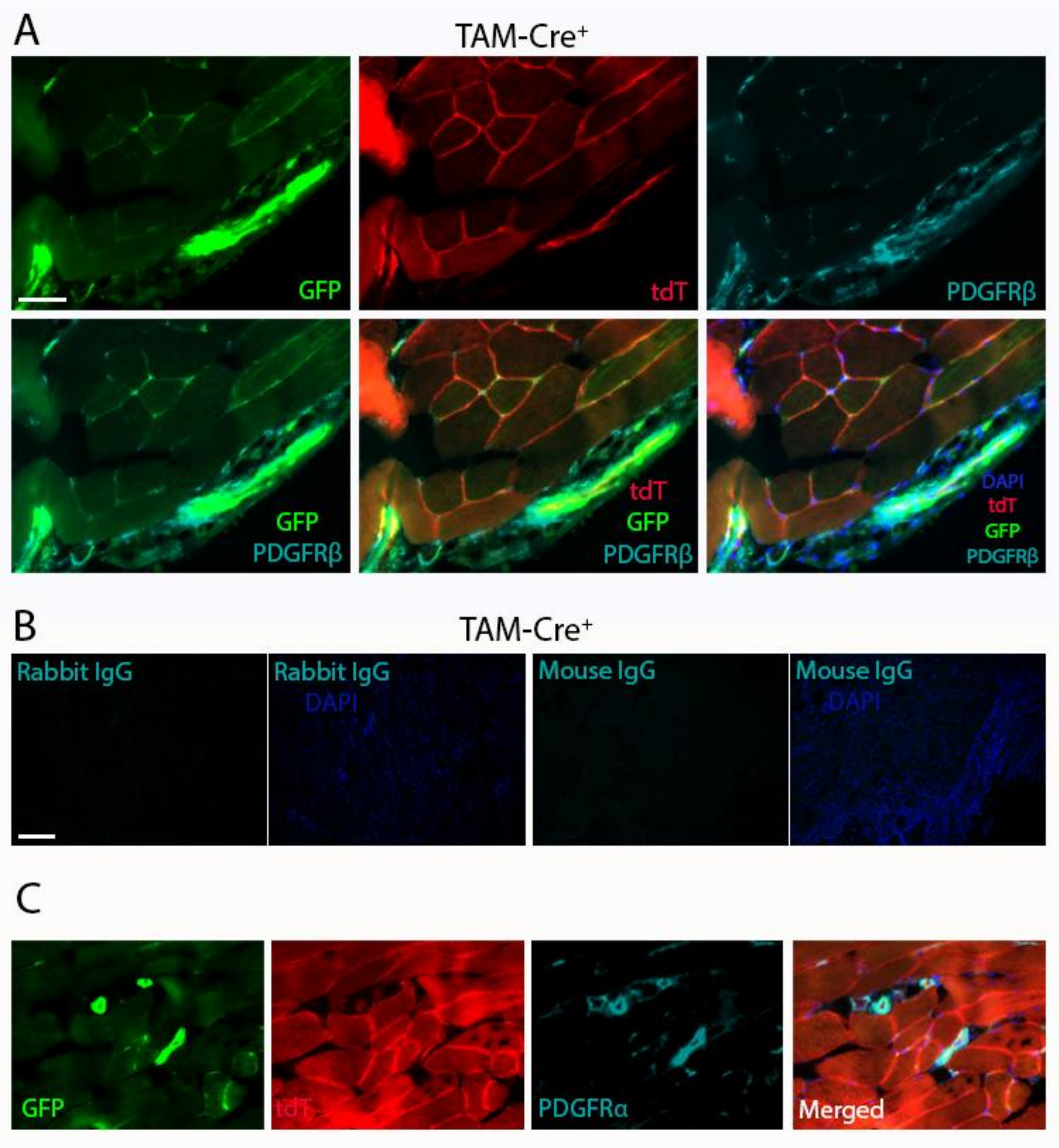

Supplementary Figure S1. TAM induced GFP expression in rotator cuff muscle PDGFRβ$^+$ cells of Pdgfrβ-CreER$^{+/-}$;mTmG (TAM-Cre$^+$) mice. (**A**) Representative images of non-injured RC sections of TAM-Cre$^+$ mouse demonstrating either GFP signal (green) or tdT signal (red) but not both, following administration of TAM for 5 consecutive days. GFP signal but not tdT signal overlaps immunolabeling of PDGFRβ (Cyan) confirming activation of GFP expression only in PDGFRβ$^+$ cells. (**B**) Rabbit and mouse primary isotype IgG control antibodies and secondary donkey anti-mouse IgG Alexa-647 or donkey anti-rabbit IgG Alexa-647 staining excludes non-specific staining of matched isotype primary antibodies. (**C**) Separate and merged channels of GFP, tdT and PDGFRα staining. Blue nuclei staining by DAPI. Scale bars are 50 μm (**A**) and 200 μm (**B, C**).

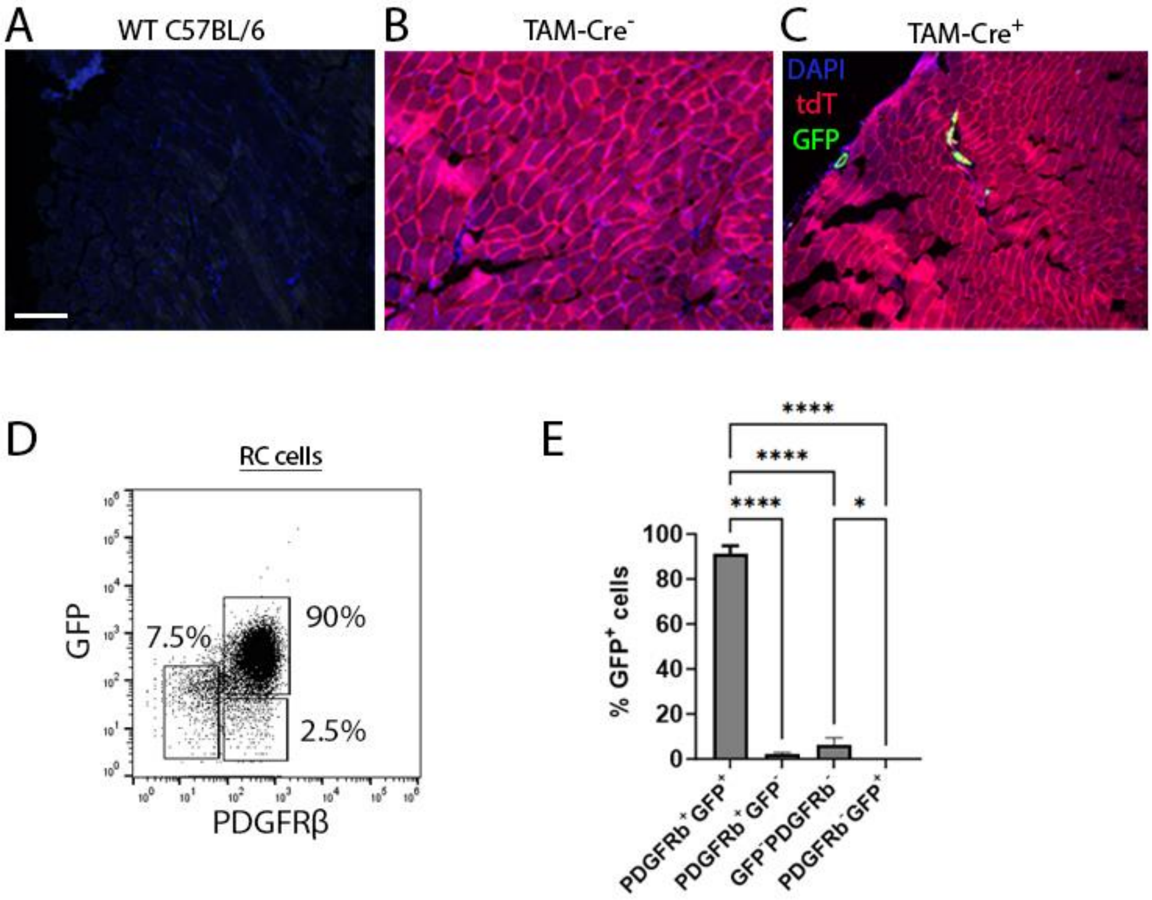

Supplementary Figure S2. Microscopic and FACS analysis of TAM induced GFP expression in non-injured RC muscles of Pdgfrβ-CreER$^{+/-}$;mTmG (TAM-Cre^{+}) mice. (**A-C**) Representative images of non-injured RC sections of TAM injected mouse strains. tdT and GFP are not expressed in all cell types and myofibers of wild type C57/BL6 muscle (**A**), only tdT is expressed in all cell types and myofibers of Pdgfrβ-CreER$^{-/-}$;mTmG (TAM-Cre^{-}) muscle, and tdT is expressed only in PDGRβ^{-} cells and myofibers while GFP expression is activated in PDGFRβ^{+} cells Pdgfrβ-CreER$^{+/-}$;mTmG (TAM-Cre^{+}) muscle (**C**). (**D-E**) RC cells were cultured overnight for recovery of PDGFRβ expression following enzymatic dissociation with Dispase that substantially reduces receptor expression from cell surface (data not shown). (**D**) Representative dot plot analysis of GFP/PDGFRβ RC cell subsets following O/N incubation. (**E**) Frequency of GFP/PDGFRβ subsets out of RC GFP^{+} cells. The absence of PDGFRβ^{-}GFP^{+} subset further validates the usability of TAM-induced Pdgfrβ-CreER;mTmG mice to study the role of PDGFRβ lineage in RC skeletal muscle remodeling. Data (mean±SEM, n = 4 mice) analyzed via one-way ANOVA and Šídák's post-hoc multiple comparisons. * $P < 0.05$, **** $P < 0.0001$. Scale bar is 200 µm.

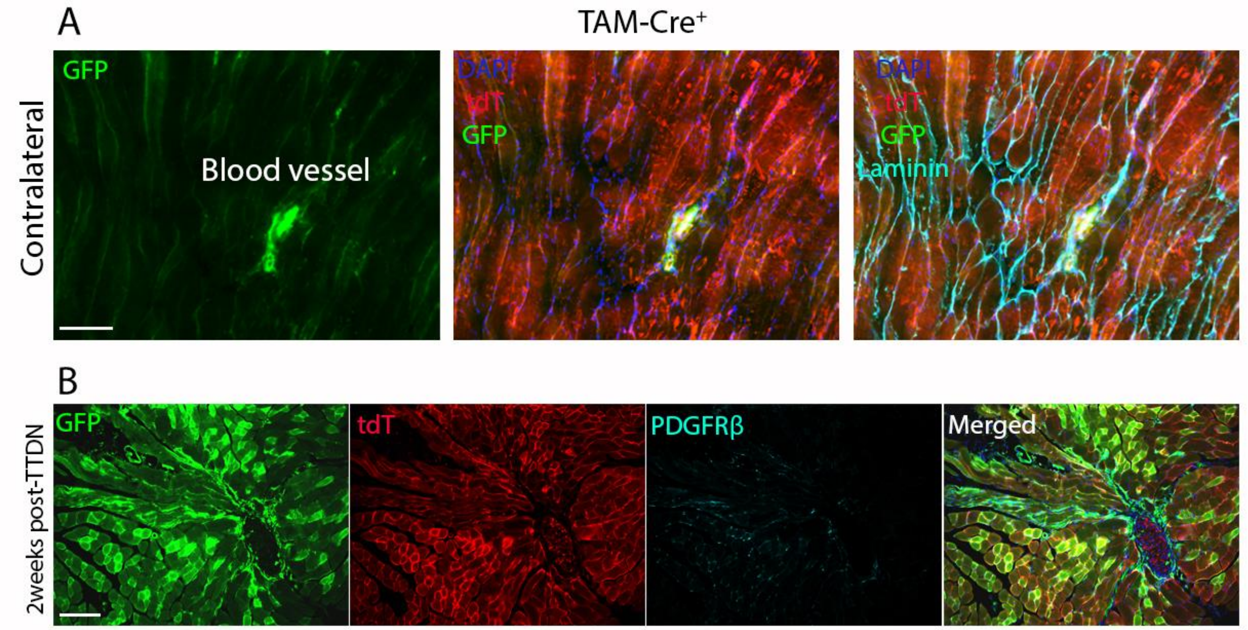

Supplementary Figure S3. Single and merged fluorescent channels of images representing contralateral RC muscle and RC muscle at 2 weeks post-TTDN. (**A**) All myofibers of the contralateral RC muscle maintained tdT signal. GFP signal is seen in blood vessel and stromal cells. (**B**) Contribution of GFP^+ cells to myofiber regeneration is seen at 2-week injured RC. Myofibers do not express PDGFRβ. Background signal was set as threshold signal for GFP (**A**, left panel) and any signal that exceeded GFP threshold was considered GFP positive. All shades of yellow myofibers are defined as mixed GFP^+tdT^+ regenerated myofibers (**B**, right panel). Blue nuclei staining by DAPI. Scale bars are 50 μm (**A**) and 100 μm (**B**).

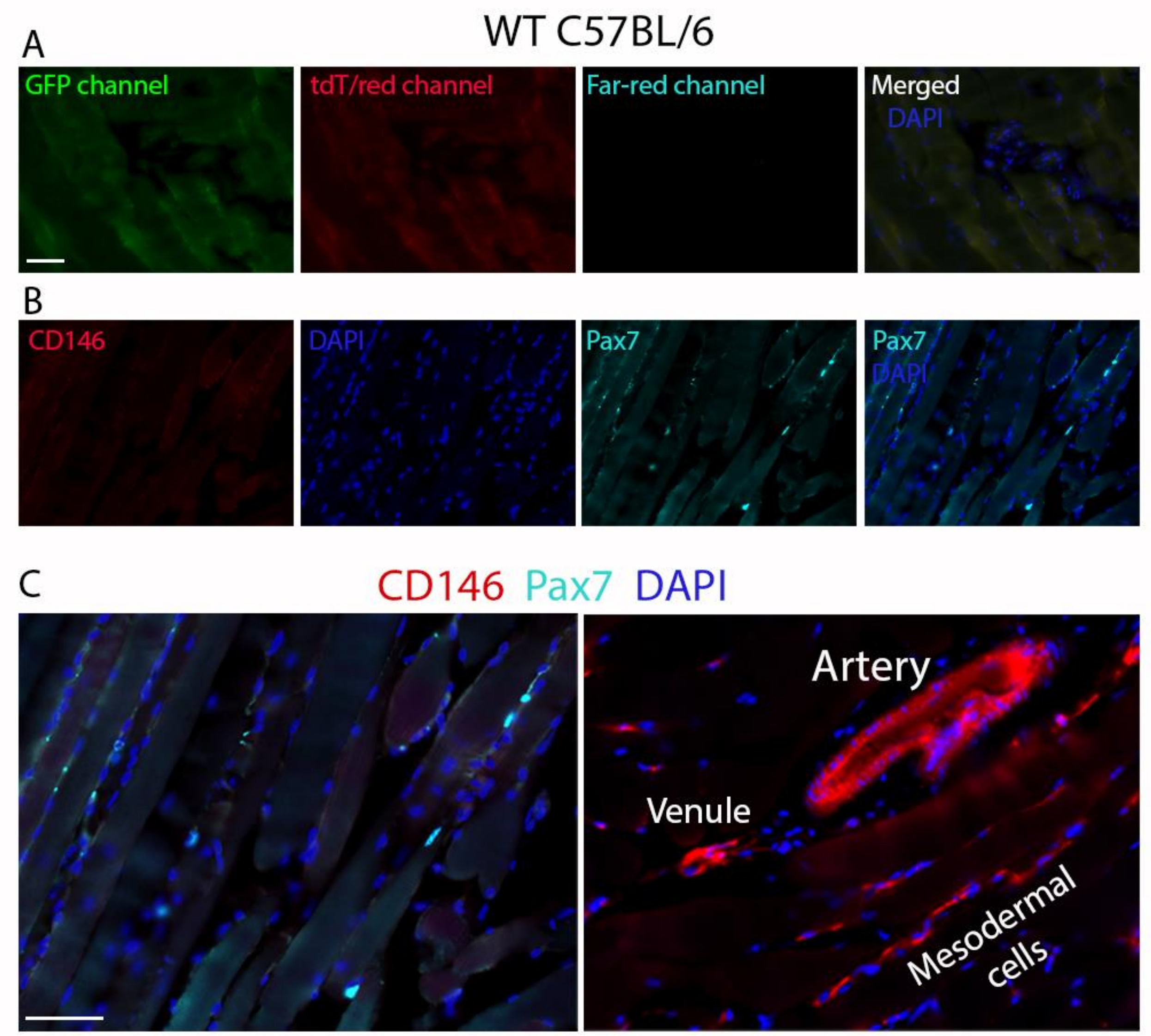

Supplementary Figure S4. Microscopic analysis of the expression of CD146 and Pax7 in sections of non-injured RC of wild type C57BL/6 mice. (**A**) Only fluorescent background is seen in representative images of sections of non-injured RC of C57BL/6 wild type mice stained with isotype IgG control antibodies. Red and far-red matched secondary antibody Alexa-fluor conjugates were used for detection of non-specific staining. GFP channel represents background fluorescence. Non-specific signal was not detected (n = 3 mice, 4 sections per RC). (**B-C**) C57BL/6 RC sections were co-stained with Pax7 and CD146 antibodies. CD146 (red) immunoreactivity was not detected in Pax7$^+$ (cyan) SC in non-injured RC. In comparison to lack of expression of CD146 (red) in Pax7$^+$ myogenic cells (**B** and **C**, left panel), high expression of CD146 is detected in artery, venule, and Pax7-negative mesodermal cells (**C**, right panel). Blue nuclei staining by DAPI. Scales bars are 100 μm (**A, B**) and 50 μm (**C**).
